# Proteomics of neurodegenerative diseases: analysis of human post‐mortem brain

**DOI:** 10.1111/jnc.14603

**Published:** 2018-11-22

**Authors:** K. W. Li, Andrea B. Ganz, August B. Smit

**Affiliations:** ^1^ Department of Molecular and Cellular Neurobiology Center for Neurogenomics and Cognitive Research Amsterdam Neuroscience Vrije Universiteit Amsterdam The Netherlands

**Keywords:** Alzheimer's disease, brain, dementia, mass spectrometer, neuroproteomics

## Abstract

Dementias are prevalent brain disorders in the aged population. Dementias pose major socio‐medical burden, but currently there is no cure available. Novel proteomics approaches hold promise to identify alterations of the brain proteome that could provide clues on disease etiology, and identify candidate proteins to develop further as a biomarker. In this review, we focus on recent proteomics findings from brains affected with Alzheimer's Disease, Parkinson Disease Dementia, Frontotemporal Dementia, and Amyotrophic Lateral Sclerosis. These studies confirmed known cellular changes, and in addition identified novel proteins that may underlie distinct aspects of the diseases.

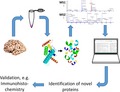

https://onlinelibrary.wiley.com/page/journal/14714159/homepage/virtual_issues.htm.

## Graphical abstract

Mass spectrometry based proteomics is an ideal technology to elucidate aberrant protein expression in disease‐affected brain. In this review, we describe the recent proteomics findings from brains affected with Alzheimer's Disease, Parkinson Disease Dementia, Frontotemporal Dementia, and Amyotrophic Lateral Sclerosis. Brain is collected. Proteins are digested by trypsin, and the resulting peptides are analysed by LC‐MS/MS. Results are validated by alternative means, for example immunohistochemistry.

**Abbreviations used**
AD: Alzheimer's disease
ALS: amyotrophic lateral sclerosis
Aβ: amyloid beta protein
CAA: cerebral amyloid angiopathy
CV: median coefficient of variation
DDA: data dependent acquisition
DLB: dementia with Lewy bodies
ECM: extracellular matrix proteins
FTD: frontotemporal dementia
LC‐MS/MS: liquid chromatography coupled to tandem mass spectrometry
LMD: laser microdissection
NFTs: neurofibrillary tangles
PDD: Parkinson disease dementia
rpAD: rapid AD

Dementias are prevalent brain disorders in the aged population. These collectively affect more than 50 million people worldwide, with an estimated economic cost of one trillion US$ in 2018 (World Alzheimer Report 2015). This major health problem will further escalate due to the increase in life expectancy. Dementia is the consequence of neurodegeneration in the brain, which develops slowly and gradually worsens over many years. Despite extensive research and clinical trials in the past decades, there is no cure for dementia, no large‐scale early diagnostic test is available for the disorders, and crucial mechanistic causes remain unclear or under debate.

There are hundreds of described neurodegenerative diseases (Przedborski *et al*. 2003), the most common form is sporadic Alzheimer's disease (AD). During the slow progression of AD, patients suffer from initial memory impairments, subsequent decline in multiple cognitive domains, and eventually their complete dependence on daily caretaking. In Western Europe 40% of people at the age of 80 and above suffer from AD. Aging is considered the major risk factor of AD, with few strong genetic risk factors, notably the *APOE4* (Mishra *et al*. 2018) and *TREM2* (Sims *et al*. 2017) genotypes. These genes have been implicated in neuroinflammation through dysfunctional microglia (Krasemann *et al*. 2017), thereby influencing AD progression. Other genes that have specifically been linked to microglia‐mediated innate immunity in AD are *ABI3* and *PLCG2* (Sims *et al*. 2017). Early onset familial AD (Bird 1999) is linked to mutations in genes for amyloid precursor protein (*APP*) and *PSEN*‐1 and ‐2. In addition, genome‐wide association studies implicated a number of genes in AD including *SORL1*,* BIN1*,* CD33*,* ABCA7* (Seshadri *et al*. 2010; Wijsman *et al*. 2011; Shen and Jia 2016; Wang *et al*. 2016). In particular, distinct *SORL1* variants have been shown to have strong influence on AD risk (Holstege *et al*. 2017).

AD is not a homogenous disease type. Apart from the typical pathological manifestations of AD, such as aggregation of Amyloid beta protein (Aβ) in plaques and hyperphosphorylated Tau (*MAPT*) in neurofibrillary tangles (NFTs), brain vascular dysfunction in the form of, for example, cerebral amyloid angiopathy (CAA) (Attems *et al*. 2011) in which Aβ accumulates in the walls of the brain vasculature, may occur in a spectrum ranging from none to high severity in different AD patients. In addition, some AD patients first show non‐amnestic AD symptoms, often with early onset, and are classified as atypical AD patients (Galton *et al*. 2000).

The second most common neurodegenerative disease is Parkinson disease dementia (PDD). It affects 2–3% of the population older than 65 (Poewe *et al*. 2017) whereas the related dementia with Lewy bodies (DLB) accounts for 15% to 20% of late‐onset dementias (Campbell *et al*. 2001). Patients often suffer from mild memory impairment, together with a high burden of motor symptoms. The disease is characterised by the deposition of α‐synuclein (*SNCA*) positive inclusions, called Lewy bodies, in the substantia nigra. The cause of the disease is incompletely understood, but it is thought to result from a complex interplay of genetic and environmental factors. Onset of the disease before age 50 is rare, but multiple mutations are known to cause early‐onset Parkinson disease (Poewe *et al*. 2017).

Amyotrophic lateral sclerosis (ALS) and Frontotemporal dementia (FTD) are two other commonly occurring forms of neurodegenerative disease. ALS patients develop muscle weakness, whereas FTD patients develop cognitive dysfunction, often including behavioural changes (Ferrari *et al*. 2011). Many patients have overlapping clinical and pathological phenotypes of both FTD and ALS, suggesting shared underlying pathological mechanisms of these two seemingly distinct disorders. Multiple gene mutations have been implicated in FTD and ALS, partly overlapping with other neurodegenerative disorders, such as mutations in the *MAPT* and Progranulin (*PGRN*) gene, which can also be seen in AD, progressive supranuclear palsy and corticobasal degeneration, and TDP‐43 (*TARDBP*) mutations, which occur both in ALS and FTD. Other mutations in ALS and FTD include *SOD1*,* FUS* and c9orf72 mutations and lead to specific disease subtypes (Ferrari *et al*. 2011).

A typical pathological hallmark of neurodegenerative diseases is the accumulation of one or more distinct proteins in aggregates, as well as their distribution and spread throughout the brain. These proteins may be modified post‐translationally in the affected brains, for example the hyperphosphorylated Tau that aggregates into NFTs in the cytoplasm of neurons, and the processing of *APP* via the β‐secretase (*BACE1*), that leads to the increased formation of Aβ plaques (Serrano‐Pozo *et al*. 2011). This suggests that proteinopathies play important roles in disease progression, where the capacity of proteins to aggregate is correlated with toxicity (Rubinsztein 2006). Misfolded proteins often possess prion‐like properties and propagate into connected healthy brain tissue (Goedert *et al*. 2017; Ruiz‐Riquelme *et al*. 2018).

This observation forms the basis of current disease staging schemes, which are based on the spatio‐temporal distribution of protein aggregates in different brain regions as revealed by (immuno‐) histochemical examination of the post‐mortem brain. In AD, the main staging schemes include the Braak stage for NFTs (Braak and Braak 1991), the CERAD score for neuritic plaques (Mirra *et al*. 1991) and the Thal phase for Aβ deposition (Thal *et al*. 2000). Since the presence and severity of each of these pathologies is thought to be important for the development and severity of AD, the National Institute on Aging‐Alzheimer's Association has introduced a staging system built up of all three pathologies (Hyman *et al*. 2012). NFTs first appear in the entorhinal cortex and hippocampal formation and later during the disease also appear in the neocortex. The spread of NFTs into the temporal cortex in Braak stage III to IV often coincides with the first appearance of symptoms, and Braak stage V or VI is observed in more severe stages of the disease (Braak and Braak 1991; Imhof *et al*. 2007). Aβ plaques first appear in the neocortex and only later affect the hippocampal formation and brain stem (Thal *et al*. 2000). Aβ accumulation preferentially starts in the precunes, medial orbitofrontal and posterior cingulate cortices, which may affect brain connectivity (Palmqvist *et al*. 2017). However, the distribution of Aβ plaques does not correlate with disease severity (Nelson *et al*. 2012). Recent studies provide clues to reconcile the spatio‐temporal segregation of Aβ and Tau in the progression of AD. The pathogenic Tau molecules initially deposited in the entorhinal cortex may spread in a prion‐like manner (Kfoury *et al*. 2012) via connecting tracks such as the hippocampal cingulum bundle to the medial parietal, lateral parietal and medial prefrontal cortex (Jacobs *et al*. 2018). These are regions with heavy and early deposition of Aβ. The convergence of toxic Aβ and Tau negatively affects these brain regions, and may account for the early episodic memory loss.

For PDD, the α‐synuclein inclusions initially appear in medulla oblongata and the olfactory bulb (stage 1–2), spread to pons, midbrain and basal forebrain (stage 3–4), and finally fill up the whole neocortex (stage 5–6). However, the association between stages and clinical severity of PDD are not apparent (Jellinger 2009). It should be noted that α‐synuclein accumulations can also be observed in around 40% of all AD cases, and parkinsonism can often be seen in advanced AD patients and cases with other forms of dementia (Spires‐Jones *et al*. 2017; Umoh *et al*. 2018). Especially in older patients, such overlap of pathologies as co‐morbidities is common. How these pathologies interact, aggravate each other, or have an additive effect on the observed symptoms, is still debated (Spires‐Jones *et al*. 2017).

The majority of dementias occur sporadically with unclear etiopathogenesis. As the underlying molecular changes of the diseases are believed to be polygenic and complex, the explorative nature of the –omics technologies, which give global views of the alterations at the levels of genome/transcriptome, proteome and metabolome, hold promise to unravel the molecular pathways underlying dementias (Redensek *et al*. 2018). Furthermore, they may give clues to uncover biomarkers for diagnosis. GWAS studies implicated several gene loci that may associate with AD. GWAS studies vary greatly in methodology and sample size, and consequently identify different, but also overlapping loci (Visscher *et al*. 2017). Some loci have repeatedly been reported and validated over several years, such as *BIN1* and *CLU* (Seshadri *et al*. 2010; Wijsman *et al*. 2011; Wang *et al*. 2016), while new loci were reported recently, including epigenomic changes (Marioni *et al*. 2018) and certain variants of *SORL1* (Holstege *et al*. 2017). How these gene products contribute to the development of AD is largely unknown. Transcriptomics is the method of choice to reveal differences in gene expression, and has been applied to examine differential expression of mRNA in AD patients. However, a recent large‐scale RNAseq analysis showed that the mRNA from AD patients was of generally poorer quality, which complicated their comparative analyses with the healthy controls (Miller *et al*. 2017). Furthermore, overlap of the RNA and protein dysregulation from AD studies showed only the modest similarity (Seyfried *et al*. 2017).

## Proteomics technology

Many dementias are described as proteinopathies. As proteomics aims to reveal tissue‐ or cell‐specific proteomes both qualitatively and quantitatively, it can potentially detect post‐translational protein modifications. It is therefore thought to be an ideal technology to elucidate the aberrant protein expression in disease‐affected brain. However, proteomics analysis of dementias met limited success for years; it was hindered by the complexity of disease etiology in brain areas with specific spatio‐temporal protein expression patterns, the profound individual variation between human subjects, and the scarcity and limited availability of correctly diagnosed diseased brain tissues of high quality. The advancement of mass spectrometry technology today with fast acquisition rate, high mass accuracy, high resolution, and ultra‐sensitivity allows a proteomics workflow that approaches the whole tissue/cell proteome detection in a reasonable time frame using low amount of tissue/cells (Hosp and Mann 2017) obtained from clinically relevant human samples.

A potential confounding factor for the interpretation of the data is the effect of post‐mortem delay time on brain tissue. A recent study revealed that none to only a few genes in human cerebellum and cortex, respectively, showed a changed expression within a few hours of post‐mortem cold ischaemia interval (Ferreira *et al*. 2018). In addition, cytoskeletal and synaptic proteins in mouse brain were shown to be generally stable up to 6 h post‐mortem delay, whereas at 24 h post‐mortem delay many of these were partially degraded (ElHajj *et al*. 2016). It is expected that short post‐mortem interval of a few hours, which nowadays is becoming the desirable norm for the collection of brain autopsies, should generally improve the analysis of brain proteomes. Thus, the collaboration of neuroproteomics researchers, neuropathologists and national and international brain banks, aids in alleviating the problem in proper sample acquisition. Together, the global changes of proteins in specific brain regions in various human dementias are starting to become elucidated.

In the present review we focus on recent publications that generally quantified thousands of proteins from which the affected cellular pathways were revealed or implicated. The prior proteomics studies (e.g., Chen *et al*. 2012; Donovan *et al*. 2012; Zhou *et al*. 2013; Musunuri *et al*. 2014; Dammer *et al*. 2015; Zelaya *et al*. 2015), and studies involving animal and cellular models, have largely been covered by previous reviews (Fasano *et al*. 2016; Moya‐Alvarado *et al*. 2016; Monti *et al*. 2018) and are not included in this review.

Proteomics is a term referring to studies interrogating the proteome of interest from biological samples qualitatively or quantitatively. Currently, the bottom‐up proteomics approach is the method of choice. It makes use of liquid chromatography coupled to tandem mass spectrometry (LC‐MS/MS) for the identification and quantification of tryptic peptides derived from the solubilised and trypsin‐digested proteins. Recent studies reported the quantification of 2000 or more proteins, and in most cases provided sufficient details to reveal the major alteration of molecular pathways in the disease‐affected brain (e.g., Hondius *et al*. 2016, 2018a) and others, see below sections for detailed description).

**Figure 1:**
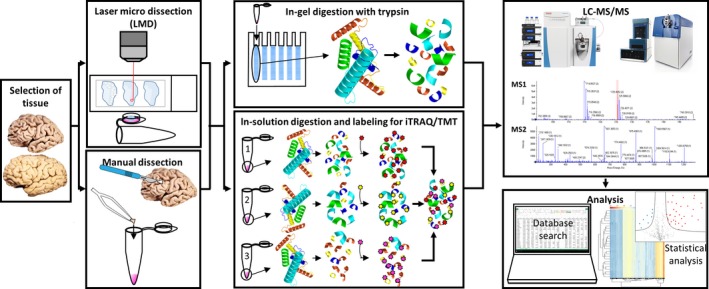
Quantitative neuroproteomics workflow. From left to right panels are the schematic representations of sample selection from Alzheimer's disease brains or heathy controls, the collection of brain areas by laser microdissection (LMD; upper panel) or manual dissection (lower panel), sample preparation for the downstream analysis by data dependent acquisition (upper panel) or isobaric multiplex labeling strategies for relative quantitative proteomics (lower panel), and the actual measurements of the peptides by LC‐MS/MS and the subsequent data analysis.

Two main quantitative proteomics approaches are often used for the analysis of biological samples, the label‐free data dependent acquisition (DDA) and the isobaric multiplex labeling strategies for relative quantitative proteomics (using reagents iTRAQ or TMT). The workflow of neuroproteomics is shown in Fig. 1. Peptides of different hydrophobicity are partially separated by reverse phase LC. All peptides eluted at a given chromatographic time are electro‐sprayed and simultaneously detected by the mass spectrometer as distinct precursor ions in the MS1 scan. The intensity of each precursor ion (peak height or peak area) correlates linearly to peptide concentration, and is used for peptide quantitation by DDA. For peptide identification, several abundant precursor ions, which can range from 5 to 40 depending on the speed and sensitivity of the mass spectrometers, are sequentially selected within a time frame of usually 1–2 s for fragmentation. A longer acquisition time per MS/MS improves detection, however, reduces the number of interrogated peptides. Subsequent database search of the fragmentation pattern on the MS2 spectrum infers the amino acid sequence of the peptide. Today, many database search engines are available as commercial packages or freeware. MaxQuant (Tyanova *et al*. 2016) is a popular freeware for academics. Alternatively, Morpheus (Wenger and Coon 2013) performs equally well with several times faster analysis time, but it only works with MS spectra generated from high resolution mass spectrometers. After a database search, the output of a large number of identified and quantified proteins should be translated into meaningful biological findings by, for example, statistical testing and clustering of functional relevant proteins. Perseus is one of the useful tools for this analysis (Tyanova and Cox 2018).

In iTRAQ/TMT experiments, all tryptic peptides from a certain sample are N‐terminally tagged by one of the eight sets of iTRAQ or the 10 sets of TMT reagents, respectively. Peptides from the next sample are tagged by the second set of iTRAQ or TMT reagents, etc. In total, peptides from a maximum of eight (iTRAQ) and 10 (TMT) samples can be separately tagged, and then pooled for LC‐MS/MS analysis. MS/MS of the peptide produces fragment ions for peptide identification. It also breaks the tags to release the signature reporter ions that are detected at low mass range and have no interference from known peptide fragment ions. The intensities of the reporter ions correlate to the peptide concentration and are used for comparative quantitation of each fragmented peptide tagged from the eight (iTRAQ) or 10 (TMT) samples.

In addition to the abundance analysis of proteomes in neurodegenerative diseases, LC‐MS/MS is also applicable for the studies of global protein post‐translational modifications including phosphorylation and glycosylation (Lassen *et al*. 2017; Wang *et al*. 2017; Ferrer *et al*. 2018) and the metabolomes (Toledo *et al*. 2017; Gill *et al*. 2018). Sample preparation and the analysis are generally more challenging than that of a typical proteomics experiment.

## Proteome changes during AD progression

It is widely accepted that neurodegenerative diseases are caused by aberrant molecular and cellular alterations in the brain that worsen over decades after onset. Nevertheless, identity of the early disease‐related processes and their development into neurodegeneration are largely unknown. This pre‐symptomatic state of AD is of great interest for designing preventive or progression‐halting treatments.

At present the earliest molecular changes documented come from the study of Hondius *et al*. (2016), who examined the protein profile changes over all AD Braak stages. The distribution of NFTs correlates with the severity of the dementia, and the topographical distribution of NFTs matches the neuropsychological profiles of typical AD. As neurofibrillary degeneration starts in the allocortex of the medial temporal lobe, the hippocampal subregions CA1 and subiculum were targeted for analysis. This defined brain region was collected by laser microdissection (LMD) from 40 cases with 5–7 cases per Braak stage. Samples were selected to not exhibit comorbidities; for example AD patients with lewy body pathology in the hippocampus were excluded. Each sample was run on an sodium dodecyl sulfate‐acrylamide gel and the gel was split into 12 slices, digested by trypsin, and LC‐MS/MS analysed in an Orbitrap Discovery mass spectrometer. Database search was performed with MaxQuant. A linear regression analysis was applied across the Braak stages at a significance cutoff level of 10% false discovery rate. In total, about 3000 proteins were identified of which 166 proteins showed higher, and 206 showed lower levels. From these 372 proteins, 89 showed significant differences in the early phase of AD between Braak stages 0‐III. Cluster analysis showed the profiles of Braak stages 0‐III and IV–VI as two major groups, suggesting major changes occurring around Braak stages III–IV.

To validate the mass spectrometric data, immunoblotting and immunohistochemical staining of selected proteins were performed. As expected, the established markers for AD, *MAPT*,* GFAP* and *CD44*, all showed profound up‐regulation that followed the Braak stages. For other proteins including *ANXA2* and *5*,* FLNA*,* VCL*,* TLN* and *TNC*, the direction and extent of fold changes in AD were consistent among these three different approaches.

The clustering analysis grouped proteins with similar expression patterns over the Braak stages that showed (i) progressive decrease, (ii) early up, late down and (iii) progressive increase. The up‐regulated group was over‐represented by astrocytic genes. This is in agreement with the increasing astrogliosis during the progression of AD (Garwood *et al*. 2017). Astrogliosis is known to contribute to inflammation that can cause damage to the brain (Ferrer 2017). Indeed, the higher expressed proteins also included those involved in response to NRF2‐mediated oxidative stress, glycolysis and cytoskeletal rearrangement. In the lower expressed protein group, neuronal proteins were over‐represented. This correlates well to the loss of synapses and eventual neuronal death. The high‐low group is suggested to reflect a compensatory mechanism that includes proteins involved in axonal guidance and oxidative phosphorylation pathways.

An alternative analysis is to examine the expression pattern of proteins that belong to the same functional group to emphasise their concomitant changes in AD progression. The extracellular matrix proteins (ECM), such as *TNC*,* VCAN*,* LAMB2* and *LGALS1*, and the adhesion molecules that interact with ECM, such as *CD44*,* ITGB1* and *ITGAV*, showed global higher expression. The increase in ECM has also been detected in an AD mouse model and has been shown to affect neuronal plasticity (Vegh *et al*. 2014).

Microtubules and associated proteins are essential for intra‐cellular transport. These proteins either showed no change (*MAPs*) or decrease (*TUBBs*). The exception was *MAPT* which increased progressively. This reflects the abundant accumulation of aberrant forms of *MAPT* in the neuron as neurofibrillary tangles. It is known that the pathologic *MAPTs* are hyper‐phosphorylated. As this study was not designed to detect phosphorylation nor to differentiate *MAPT* isoforms, it remains unclear which *MAPT* isoforms and their phosphorylation patterns were altered during progression of AD. A previous study identified seven types of tau modifications at 63 sites in mice (Morris *et al*. 2015). The glutamate receptors AMPA receptor subunits *GRIA 1*–*3* and NMDA receptor subunits *GRIN1* and *2B*) showed progressive decline. A recent study (Reinders *et al*. 2016) indicated that loss of GRIA3 may protect against Aβ‐associated memory impairment. Unlike α‐amino‐3‐hydroxy‐5‐methylisoxazole‐4‐propionate and NMDA receptors, several presynaptic proteins (*BSN*,* LIN7A*,* RIMS1* and *RAB3C*) and post‐synaptic proteins (*DLG2*‐*4*,* DLGAP1*,* HOMER1* and *SHANK3*) showed an initial up‐regulation in Braak stages I–III, followed by a decline towards Braak stage VI. On the other hand, typical vesicle integral proteins, such as *VAMP2* and *SV2A,* showed no change. Several endocytotic proteins such as *CLTA* and *AP2M1* showed reduced expression, and *SNAP91*,* AP2A1*,* AP2B1* and *DNM1* showed early decrease, suggesting an early endocytic dysfunction. The global decline of all these pre‐ and post‐synaptic proteins from stage IV onwards signals the loss of synapses, through which cognitive decline might occur.

More recently, Lachen‐Montes and colleagues (Lachen‐Montes *et al*. 2017) examined proteome changes in the olfactory bulb from each 3–5 cases of healthy controls, as well as initial, intermediate and advanced AD stages. Samples were extracted in urea lysis buffer containing CHAPS and run very briefly in an sodium dodecyl sulfate acrylamide gel. The small gel piece containing all the proteins was cut and digested using trypsin. The peptides mixtures were fractionated with a 240 min gradient of LC run and analysed in a label‐free data dependent mode using a Sciex 5600 TripleTop MS. The relatively long LC run time should allow acquisition of a higher number of MS/MS events, which should lead to an increase in number of proteins identified. From the 1311 quantified proteins across all experimental groups, 110, 125 and 159 proteins from initial, intermediate and advanced AD cases, respectively, were differentially expressed when compared to the healthy controls. Significantly, more than 50% of these proteins tend to localise to the synaptic terminal, indicating a progressive synaptic degeneration during AD progression. When network analysis was performed, the initial stages showed dysregulation in the actin based cytoskeleton and the regulators of the interaction between components of the cell‐cell junction, the intermediate stages showed impaired mitochondrial functions and imbalanced redox signaling, and the advanced stages seemed to have impaired RNA stability and pre‐mRNA splicing processes.

## Proteomes of asymptomatic and symptomatic AD

One major pathological hallmarks of AD is deposition of Aβ leading to Aβ plaques formation in the brain. However, the spatio‐temporal appearance pattern of Aβ plaques does not always correlate to the severity of the AD symptoms, especially at advanced ages (Sperling *et al*. 2011; Ganz *et al*. 2018).

To examine why individuals can have high amounts of Aβ without clinical signs of AD, Seyfried and coworkers compared 15 asymptomatic (AsymAD) and 20 symptomatic AD cases with 15 healthy controls from dorsolateral prefrontal cortex and precuneus (Seyfried *et al*. 2017). Precuneus is the first brain region with Aβ aggregation that spreads to orbitofrontal cortex and then neighbouring areas. This early Aβ accumulation is associated with hypoconnectivity within the default mode network and to the frontoparietal network (Palmqvist *et al*. 2017). At later stages the patients show glucose hypometabolism and eventually atrophy.

In the study of Seyfried, each piece of brain tissue was individually weighed (∼0.1 g) and homogenised in 500 μL of urea lysis buffer. After digestion with lysyl endopeptidase and trypsin, peptides were analysed on a Q‐Exactive plus mass spectrometer with a 2 h LC gradient acquired in DDA mode. About 5000 proteins were identified, from which 2735 protein groups which were present in 90% of samples were used for quantitation. When compared to the controls, 63 and 280 proteins from AsymAD and AD, respectively, showed significant differences. Thus, the much larger number of changes in AD correlated to the increased pathological burden.

Several proteins showed progressive changes from controls to AsymAD and AD. The ECM proteins *CSPG*,* ACAN* and *PTN* showed progressive increase, whereas proteins involved in synaptic function and synaptogenesis showed progressive decrease (*SYNPO1*,* VGF*,* CAMKK2, CAMK4)*. This is in general agreement with the proteome changes observed in the hippocampus according to the Braak stages, in which synaptic proteins showed a decrease and ECM proteins increase progressively (Hondius *et al*. 2016). *CLU* and *PEPD* were altered exclusively in the symptomatic phase of AD.

Proteins involved in a distinct biological process could be co‐regulated in different disease stages. To reveal protein co‐expression, weighted gene co‐expression network analysis was applied and identified 16 modules ranging from the biggest module of 396 proteins to the smallest 28 proteins. Several modules of expression profiles showed significant decrease in AD. They were enriched with neuronal markers or mitochondrial proteins, consistent with the hypometabolism and synapse loss observed in AD patients (Mattson 2004). A module linked to inflammatory response was increased in AsymAD. This suggests the activation and proliferation of astrocytes and microglia. Another module enriched with microglia and astrocyte markers was increased only in AD. When the risk loci based on the GWAS data were used, the AD candidate genes were over‐represented in a module enriched in oligodendrocyte, and a module in astrocyte and microglia.

Together, the studies of Seyfried and Hondius (Hondius *et al*. 2016) reveal similarities in the alteration of functional protein groups in AD; both point to the increase of glial cell protein networks and the decrease in specific neuronal proteins as important factors that probably lead to cognitive and memory impairment.

## Region specific protein expression in AD

It is clear that different brain regions develop different AD pathologies at different times. To reveal these spatial changes in the brain, Xu and colleagues (Xu *et al*. 2018) reported the proteomics analysis of six brain regions from controls and AD patients. The selected regions were grouped as heavily affected (hippocampus, entorhinal cortex and cingulate gyrus), lightly affected (sensory cortex and motor cortex), and the least affected cerebellum that lacked NFTs and showed only low numbers of Aβ Plaques. 8‐plex iTRAQ technology was used for quantitative proteomics analysis. A total of 4825 proteins were quantified in at least one brain region, and 1899 proteins were assigned to all six regions.

As expected, the heavily affected regions showed the highest number of protein level changes of around 30%, whereas the lightly affected regions have only 11–13% abundance changes. Unsupervised clustering analysis demonstrated a similar pattern of expression changes for these five regions, and that the lightly affected regions undergo molecular alterations equivalent to those in earlier stage of AD in the heavily affected regions. Interestingly, the supposedly spared cerebellum registered a relatively large change of 20%, with a profile distinct from the other five regions. The most consistent changes across all brain regions were the proteins and pathways involved in the immune response. Proteins involved in apoptosis and cell cycle regulation were dysregulated in severely affected regions. Metabolic impairments were also observed. Correlation network analysis identified four proteins with the most overall influence on AD pathogenesis (*STXBP1*,* CRMP1*,* ACTR10* and *AMPH*). This points to the importance of neurotransmission processes. This study also recapitulated the molecular changes observed in hippocampus and precuneus.

The protein changes observed in the cerebellum were distinct from those in other regions. The cerebellum showed stronger reduction in the abundance of proteins involved in the electron transport chain and an increase in oxidative defense proteins. Together, they may provide a protective mechanism that decreases ROS‐production while increase ROS defenses. Furthermore, the Purine ribonucleosides degradation pathway was activated. Therefore, it is proposed that the cerebellum actively induces a unique pattern of up‐regulated neuronal survival pathways alongside protection against oxidative and inflammatory damage, which protects the cerebellum from degeneration.

## Proteome from AD patients with cerebral amyloid angiopathy

CAA involves the deposition of amyloid in the brain vasculature. Patients with CAA have an increased risk of cerebral infarction, cerebral haemorrhage and micro‐bleeds. There are several types of CAA based on the amyloid protein involved (Yamada 2015). Among them, the sporadic Aβ type CAA is often detected in up to 80% of all AD patients. CAA occurs as two distinct types. CAA‐type 2 refers to cases where deposition of Aβ is present only in larger blood vessels, which include leptomeningeal vessels, cortical arteries and arterioles. In CAA‐type 1 cases, brain capillaries are also affected (Yamada 2015). In some AD cases, CAA type‐1 appeared to be the primary cause of rapid progressive dementia.

To examine the pathological differences of Aβ deposition in parenchyma and those related to the vasculature, Hondius and colleagues performed a discovery proteomics analysis on laser capture microdissected tissues of the occipital tissues from six cases of healthy controls without any Aβ or tau pathologies, seven cases of AD with severe plaque pathology but no vascular deposits, and seven cases of AD with severe CAA‐type 1 pathology and a negligible amount of plaque pathology (Hondius *et al*. 2018a). The occipital lobe was chosen because it is the brain region most heavily affected with CAA pathology. Grey matter enriched for Aβ aggregates was collected for analysis. The selected tissue areas have high plaque load in the AD cases, and contained many affected capillaries and larger vessels in the CAA cases. The same anatomical regions were isolated from control cases. More than 2000 proteins were identified in total. The authors took an exploratory approach with relaxed tests aimed to discover CAA‐type 1 specific proteins, while allowing a higher rate of false positives that can subsequently be falsified by independent focused studies. For example, a protein should be present in most CAA cases but at most once in AD and/or control cases, and vice versa. Nine proteins fulfilled this criterion (*HLA*‐*DRA/DQA2*,* HTRA1*,* APCS, COL6A2, MOB2, POTE1, KIAA1468, TMF1* and *SGIP1)*. For most other proteins, a simple *t*‐test with *p* < 0.05 was applied, which yielded 20 proteins (*CLU, APOE, SUCLG2, PPP2R4, KTN1, ACTG1, TNR, COL6A3, NFASC, APP, UBLCP1, SR1, NDP, PNP, C1orf123, DHX15, SYNPO, TPM1, CADPS2* and *SERPINA3*).

Based on the fold change or specific expression in the CAA group compared to the AD and control groups, several proteins were selected for validation. *APOE, NDP, APCS* and *COL6A2* all showed the same trend of an increased abundance in the CAA group. Immunohistochemistry subsequently confirmed the MS results. For example, *NDP* was abundantly present around the vasculature in the CAA cases. For cases that have dysphoric Aβ, diffuse staining of *NDP* was found in the parenchyma. *NDP* was not detectable in controls. When IHC was quantified, *NDP* and *COL6A2* showed strongly increased immmunoreactivity specifically in CAA cases, and moderate increase for *APCS, HTRA1* and *APOE*.

IHC revealed that the CAA specific proteins are also present in other brain vascular defects. In short, *COL6A2* appeared as a general small vessel disease marker. *NDP, APOE* and *APCS* were abundant in CAA and disease with cotton wool plaque pathology, as well as prion CAA. They were present in lower level in cerebral autosomal dominant arteriopathy with subcortical infarcts and leucoencephalopathy (CADASIL). In particular, the study suggested *NDP* as a useful marker to separate CAA from Aβ plaque pathology.

Two recent studies reported the proteomics of CAA‐type 2. As leptomeningeal and cortical vessels are large and easily distinguished from other structures, they were either removed from the brain with a micro‐scalpel or dissected via LMD. Inoue and coworkers performed proteomics on eight cases of severe CAA, 12 cases of mild CAA and 10 controls (Inoue *et al*. 2017), 60% of the CAA cases and 15% of control cases had Braak stages of three and above. It is stated that six proteins were significantly higher expressed in severe CAA, i.e., Aβ, *APOE*,* GFAP*,* ENO 1/3*, and *SRPX1*. IHC revealed the co‐accumulation of *SRPX1* and Aβ in cerebral blood vessels. A subsequent functional study indicated that *SRPX1* may increase Aβ‐induced cerebrovascular degeneration in CAA. Manousopoulou and coworkers collected five cases of young controls, seven cases of elderly controls and five cases of severe CAA (Manousopoulou *et al*. 2017). The iTRAQ technology was chosen for quantitative proteomics analysis. The caveat is that a maximum of eight samples can be processed in parallel per analysis. Therefore, two young and two elderly controls and four CAA cases were randomly selected for the proteomics analysis. With this low number of replications, data analysis to reveal differential expression of proteins is challenging. The authors reported a large number of proteins that show differences among the young and elderly controls and the CAA cases. IHC revealed the co‐localisation of *CLU* with Aβ within the walls of leptomeningeal arteries in the CAA cases. *TIMP2* staining was found to be restricted to walls of arteries, and increased in CAA. In a separate study using a mouse model (Wojtas *et al*. 2017), *CLU* was suggested to function as a major Aβ chaperone to maintain Aβ solubility along interstitial fluid drainage pathways and prevent CAA formation.

## Analysis of amyloid plaques distinguish rapidly progressive and sporadic AD

In a small population of AD patients the disorder progress rapidly, with a median survival time of 7–10 months (called rapid AD; rpAD). This is in contrast to the typical sporadic AD, which develops slowly for years. The rpAD patients do not have pathogenic mutations in *APP* or *PSEN1/2*. The pathological hallmarks of Aβ plaque and neurofibrillary tangle are comparable between the rapidly progressive AD and the sporadic patients.

To better understand rpAD, Drummond and colleagues compared the proteomes of amyloid plaques isolated from 22 cases each of rpAD and sporadic AD (Drummond *et al*. 2017). The immunohistochemically stained plaques from formalin‐fixed paraffin‐embedded tissue blocks were selected and microdissected via LMD and about 740 plaques per sample were collected. The tryptic peptides were analysed with a Q‐Exactive MS using a 2 h LC‐gradient in DDA mode. About 900 proteins were identified per sample, in which 279 proteins were consistently detected from every case, including the typical amyloid plaque proteins Aβ, *APOE*, tau, *GFAP* and *CLU*. When compared to sporadic AD cases, the rpAD cases have 85 proteins with increased expression (for example *SNCA*) and 56 with decreased (for example Aβ, *GSN* and *GFAP*) expression. Some other important plaque‐associated proteins (for example tau, ubiquitin and *APOE*) were similarly expressed. The rpAD plaques contained significantly higher level of neuronal proteins and lower levels of astrocyte proteins. Pathway analysis suggested an increase in neurotransmission related proteins. It was proposed that the altered process of synaptic vesicle release may play a role in plaque formation, which may then contribute to the rapid disease progression.

## Proteomics analysis of other dementias

AD and Lewy body dementia are the most common forms of neurodegenerative diseases. Bereczki and colleagues compared the prefrontal cortex proteomes from 32 cases (eight controls, eight PDD, seven DLB and nine AD) (Bereczki *et al*. 2018). Peptides from each sample were labelled with TMT 10‐plex reagents. After pooling eight samples and two internal control samples, peptides were fractionated by isoelectric focusing on an IPG strip that separated peptides according to their charges. 72 fractions were recovered from the IPG strip and analysed by LC‐MS/MS separately. The data were acquired on a Q‐Exactive mass spectrometer with a 74 min LC gradient. In total 10 325 proteins were identified, of which 7033 proteins were common to all 32 samples. When compared to controls, there were 1010 differentially expressed proteins in DLB, 485 in PDD, and 593 in AD. Among the 851 proteins related to synaptic transmission, 25 were significantly altered in these dementias including presynaptic proteins (*GRIK2, CAMK2A, BDNF, PDYN*), synaptic vesicle priming proteins (*SNAP47*), synaptic vesicle proteins (*SV2, SYT2*), proteins found in both pre‐ and post‐synapse (*GAP43, LRFN2*) and post‐synaptic proteins (*GRIA3, GRIA4, ARC, CNIH2, PVRL3, NRGN*). As this study focused on the synaptic markers of neurodegenerative diseases, the bulk of other regulated proteins that may give clues to disease mechanisms remains to be interrogated in detail.

To differentiate the differences between PDD and DLB, Datta and coworkers performed an iTRAQ experiment on two replicates of pooled samples from BA9 area from patients of PDD, DLB and controls (Datta *et al*. 2017). The lysed tissues were trypsin digested, labelled with iTRAQ, fractionated by anion‐exchange chromatography into 20 fractions. Each fraction was analysed by LC‐MS/MS on a QStar mass spectrometer with a 90 min LC gradient. A total of 1914 proteins were identified, but none of the proteins showed a significant and opposite regulation between DLB and PDD when compared to controls. The authors contributed the similarity of DLB and PDD to the amyloid burden, which is a common co‐morbidity, especially in the elderly cases (Spires‐Jones *et al*. 2017).

ALS and FTD are clinically distinct diseases, yet many ALS patients also develop FTD, and vice versa, suggesting common disease mechanisms. Umoh and coworkers compared the frontal cortex proteome changes from patients of ALS, FTD, ALS/FTD, and healthy controls (Umoh *et al*. 2018). Protein co‐expression network analysis revealed 15 modules. The modules showed that proteins with increased expression in FTD were enriched for RNA splicing, response to biotic stimuli, zinc ion binding, homeostatic processes and blood microparticles and circulation immunoglobulin complexes. The latter process implicates damage of the blood‐brain barrier. The decreased modules include synaptic and neuronal proteins and mitochondrial proteins. The protein expression for ALS cases was similar to the controls. This is consistent with the fact that TDP‐43 pathology is not found in the frontal cortex, and that there is no cognitive decline. For the ALS/FTD group, the changes in the modules were the intermediate between ALS and FTD groups.

## Proteomics studies of fluid biomarkers for neurodegenerative diseases

The current diagnostic tests for AD and FTD are dominated by the identification and quantification of Aβ, tau or phospho‐tau in the cerebrospinal fluid (CSF) (Blennow and Zetterberg 2018; Mattsson *et al*. 2018), which reflect the plaque and tangle pathologies in the brains of patients. However, co‐pathologies are common in dementia and cannot be distinguished by measuring Aβ/tau alone. Additional biomarkers should be included to improve specificity. In the past decade, studies on biomarkers in CSF focused mainly on one or a few proteins, and only few studies took an explorative proteomics approach (Portelius *et al*. 2017). While a few potential biomarkers have been proposed, none have reached clinical use as diagnostic tool.

A label free MS quantitation has been carried out on CSF of FTD patients with TDP‐43 (*n* = 12) or tau (*n* = 8) pathology, and cases with subjective memory complaints (*n* = 10) as controls (Teunissen *et al*. 2016). More than 1900 proteins were identified in total, of which 56 proteins were considered differentially regulated between groups. ELISA assays were performed on a small set of these proteins, namely *FABP4*,* YKL*‐40, complement factor D, *IL1RAP* and *APOL1*, where only *YKL*‐40 could be validated to exhibit higher levels in FTD cases than controls. *YKL*‐40 has been found to be increased in different neurodegenerative diseases (Alcolea *et al*. 2014), and may reflect the abnormality of astrocyte activity associated with inflammatory processes (Llorens *et al*. 2017).

The comparison of CSF proteomes of AD and controls was reported by Khoonsari *et al*. (Khoonsari *et al*. 2016). About 200–300 proteins were identified, many of which showed differences between the AD and control groups. Eight proteins (*A2GL*,* APOM*,* C1QB*,* C1QC*,* C1s*,* FBLN3*,* PTPRZ* and *SEZ6*) were further verified by an antibody‐based method to have lower levels in AD cases.

## Future perspectives in neuroproteomics

A typical brain tissue sample contains thousands proteins, and an even larger number of isoforms (proteoforms) generated from alternative splicing of a gene or post‐translational modifications of the protein. A pre‐fractionation of tryptic peptides in conjunction with the use of a state‐of‐the‐art mass spectrometer, such as the Obitrap HF‐X with 40 Hz scanning speed (Kelstrup *et al*. 2018) or the ion mobility Q‐Top mass spectrometer (Tims TOF pro) equipped with PASEF technology (Meier *et al*. 2015), enable the identification of over 10 000 protein groups and tens of thousands of phosphopeptides by data dependent acquisition (Bekker‐Jensen *et al*. 2017). These most advanced proteomic pipelines rival the coverage generally achievable by transcriptomics. This degree of coverage, however, should be balanced by the analysis time, the reproducibility across multiple fractions from single sample, and the high cost and availability of the advanced mass spectrometers. Alternatively, data independent acquisition (SWATH) holds promise to interrogate several thousand proteins in 1–2 h of analysis time from commonly used research grade mass spectrometers (Kelstrup *et al*. 2018; Koopmans *et al*. 2018). This technique has the potential to develop into a high throughput methodology and is particularly useful for experiments of large sample sizes, such as the analysis of multiple brain regions across different neurodegenerative stages from various dementias.

In addition to the mass spectrometry itself, also the auxiliary technology, such as LMD as a mean to get to single cell type resolution in proteomics analysis, are being developed. Specific mass spectrometry compatible staining protocols (Hondius *et al*. 2018b) are contributing tremendously to the use of mass spectrometry in neurodegenerative disease.

## Conclusion

LC‐MS/MS is the driving force of proteomics studies. With the current advancement of mass spectrometers in sensitivity and speed, it is now possible to interrogate thousands of proteins from whole tissue lysates. The application of quantitative bottom‐up proteomics in the past few years is starting to reveal the alteration of proteins and their networks during progression of neurodegeneration, and suggest candidate proteins as potential biomarkers for specific neurodegenerative diseases.
